# The role of miR-557 in modulating cervical cancer malignancy by targeting TAOK1

**DOI:** 10.1186/s41065-026-00695-6

**Published:** 2026-06-12

**Authors:** Yilin Yin, Yingying Huang, Li Lin, Mingxiu Cui, Linge Zhong

**Affiliations:** 1 Shanghai Medical College of Fudan University, Shanghai, 200032 China; 2 People’s Hospital of Yangjiang, Guangdong, 529500 China; 3 Department of Gynecology, The People’s Hospital of Longhua, Shenzhen, Shenzhen, 518100 China; 4 Department of gynaecology and obstetrics, Nanjing LuHe People’s Hospital, Nanjing, 211500 China; 5 Department of Obstetrics and Gynecology, Shanghai Sixth People’s Hospital Affiliated to Shanghai Jiao Tong University School of Medicine, No. 222, Huanhu West rd Road, Nanhui New Town, Pudong New District, Shanghai, 201306 China

**Keywords:** miR-557, TAOK1, Cervical cancer, Proliferation, Biomarker

## Abstract

**Background:**

Cervical cancer (CC) remains a major threat to women’s health globally. Although miR-557 is a tumor suppressor in multiple cancers, its role in CC remains unclear.

**Objective:**

This study investigated whether miR-557 regulates CC progression by targeting TAOK1.

**Methods:**

This study collected 113 pairs of cervical cancer tissues and adjacent tissues. The expression of miR-557 was detected by qRT-PCR, and its relationship with clinicopathological features and prognosis was analyzed. In HeLa and SiHa cell lines, we performed functional assays (CCK-8, Transwell) and measured ferroptosis-related indicators (Fe²⁺, GSH, GSH-Px) after transfecting miR-557 mimic or TAOK1 overexpression vectors. The miR-557/TAOK1 targeting relationship was validated by dual-luciferase reporter assay and rescue experiments.

**Results:**

miR-557 was significantly downregulated in CC tissues and cell lines. Low miR-557 expression correlated with advanced FIGO stage, LNM, and poor prognosis. Functionally, miR-557 overexpression inhibited CC cell proliferation, migration, and invasion, accompanied by increased Fe²⁺ levels and decreased GSH and GSH-Px activity. TAOK1 was identified as a direct target of miR-557, and its overexpression reversed the tumor-suppressive effects and ferroptosis-related changes induced by miR-557.

**Conclusions:**

miR-557 is significantly downregulated in CC and exerts tumor-suppressive functions. By targeting TAOK1, it inhibits the malignant progression of CC cells and may act as a promising biomarker for CC.

## Introduction

Cervical cancer (CC) remains a leading malignancy among women worldwide, with persistently high incidence and mortality rates, particularly in developing regions with limited medical resources [1, 2]. Although human papillomavirus screening and preventive vaccination have been implemented in most countries, the prevention and treatment of CC still face severe challenges due to the uneven distribution of medical resources and lagging public awareness [3]. Moreover, current mainstream early diagnostic methods have limitations in sensitivity and specificity, making it difficult to fully meet the demands of precision screening [1]. Therefore, identifying novel biomarkers has become a key priority for enhancing early diagnosis and prognosis assessment of CC.

MicroRNAs (miRNAs) are endogenous short non-coding RNAs that post-transcriptionally regulate gene expression [4]. With the in-depth study of non-coding RNAs, the abnormal expression of miRNAs in CC and their regulatory networks have become research focuses in the field. Notably, a previous study reported that miR-557 expression was significantly decreased in CC tissues [5], suggesting its potential involvement in CC progression. Consistently, multiple lines of evidence have indicated that miR-557 shows significantly reduced expression in various malignant tumors, including pancreatic cancer [6], hepatocellular carcinoma [7], and lung cancer [8]. By targeting and regulating key genes, it exerts tumor-suppressive effects and is thus recognized as a critical tumor suppressor. For instance, in pancreatic cancer, miR-557 inhibits proliferation and invasion by targeting EGFR [6]; in hepatocellular carcinoma, it suppresses progression via the Wnt/β-catenin pathway by targeting RAB10 [7]; and in lung cancer, it functions as a tumor suppressor by negatively regulating LEF1 [8]. However, despite its downregulation in CC having been observed, the functional role and molecular mechanism of miR-557 in cervical cancer remain poorly understood. Therefore, this study aimed to investigate the regulatory role and underlying mechanism of miR-557 in CC.

TAOK1 functions as a critical signaling regulator in tumor initiation and progression [9]. Research has indicated that TAOK1 exhibits significant oncogenic effects across various cancers, including non-small cell lung cancer [10] and hepatocellular carcinoma [11]. Importantly, a multi-omics analysis showed that TAOK1 is generally upregulated in 12 cancer types. Additionally, the study confirmed significant upregulation of TAOK1 in CC SiHa cells, where its overexpression directly promotes cell proliferation, thereby driving disease progression [12]. Using the TargetScan database, TAOK1 was preliminarily identified as a candidate downstream target gene of miR-557. Based on these findings, we hypothesized that a potential regulatory relationship may exist between miR-557 and TAOK1 in CC; however, the specific mechanism requires further validation.

This study aims to investigate the regulatory role and molecular mechanisms of miR-557 in CC, including the clinical significance and predictive value of its aberrant expression in CC. Furthermore, it seeks to elucidate the mechanism by which miR-557 regulates the malignant phenotypes of CC cells by targeting TAOK1. This study is expected to provide novel biomarkers and a theoretical foundation for the prognosis assessment and targeted therapy of CC.

## Patients and methods

### Patient samples

This study included 113 patients diagnosed with CC who underwent examination at Nanjing LuHe People’s Hospital between January 2017 and December 2019. Paired tumor and adjacent normal tissue samples were collected from each patient during surgery. This study was approved by the Medical Ethics Committee of Nanjing LuHe People’s Hospital and proceeded after all participants provided written informed consent.

*Inclusion criteria*: (1) Patients with CC who have been pathologically confirmed and clinically staged as FIGO I-III; (2) Patients have not received any prior treatment; (3) Patients’ clinical data were complete; (4) Patients have good compliance and are able to cooperate in completing the examination.

*Exclusion criteria*: (1) Patients with a history of other primary malignant tumors; (2) Patients who are in pregnancy or lactation; (3) Patients with severe liver or kidney disease; (4) Patients with other gynecological disorders.

### Follow-up

Postoperative follow-up was conducted for all patients over a period of 5 years. The follow-up period was calculated from the date of surgery, with the endpoint being either the patients’ death or the completion of the 5-year follow-up period. Follow-up data were collected through multiple channels, including outpatient follow-up visits, telephone consultations, and online electronic medical record systems.

### Cell culture

CC cells including HeLa (WN-C20463), SiHa (WN-C20311), CaSki (WN-C20195), MS751 (WN-C20184), and C-33 A (WN-C20183), as well as immortalized human cervical epithelial cells HCerEpiCs (WN-01978), were all purchased from Wuhan Hualina Biotechnology Co., Ltd. (China). Cells were inoculated in MEM medium containing 10% Fetal Bovine Serum (FBS, 164250, Wuhan Pricella Biotechnology Co., Ltd., China) and cultured in a constant temperature incubator (3111, Thermo, USA) containing 5% CO_2_ at 37 ℃.

### Cell transfection

miR-557 mimics, inhibitors, TAOK1 overexpression vector (oe-TAOK1), and their corresponding negative controls (NCs) were all obtained from Sangon Biotech (Shanghai, China). Cells in the logarithmic growth phase were collected, the cell density was adjusted to 5 × 10⁵ cells/mL, and the cells were seeded into 6-well plates. Transfection was initiated when cell confluence reached 80%. The transfection was performed using Lipofectamine 3000 transfection reagent (L3000015, Invitrogen, USA). After 6 h of transfection, the medium was replaced with complete medium containing 10% FBS and the cells were further cultured.

### qRT-PCR

Total RNA was extracted from tissues and cells by TRIzol reagent (15596026, Invitrogen, USA). Total RNA was reverse transcribed into cDNA using the PrimeScript RT Kit (RR037A, TaKaRa, Japan). Using cDNA as a template, amplification reactions were performed on a real-time fluorescent quantitative PCR instrument with SYBR Green qPCR Master Mix (4309155, Thermo, USA). U6 was used as the reference gene for miR-557, and GAPDH was used as the reference gene for TAOK1. The relative expression level of the target gene was calculated using the 2^−ΔΔCt^ method.

### CCK-8 assay

Cell proliferation activity was detected using the CCK-8 method. The cell density was adjusted to 5 × 10^5^ cells/mL and the cells were inoculated into a 96-well plate. Then, 10 µL of CCK-8 reagent (C0038, Beyotime, Shanghai) was added to each well at 0, 24, 48 and 72 h respectively and the plate was then incubated in a dark chamber for 4 h. The absorbance of each well was detected using an enzyme-linked immunosorbent assay plate reader (iMark, Bio-Rad, USA) at a wavelength of 450 nm, and the cell viability was calculated.

### Transwell assay

Cells in the logarithmic growth phase were collected, resuspended in serum-free medium, and counted. The cell suspension was added to the upper chamber, and the complete medium containing 10% FBS was added to the lower chamber. The invasion experiment needed to be pre-performed by coating the bottom of the upper chamber with Matrigel (356234, Corning, USA), and then incubated at 37 ℃ to allow it to polymerize. Placed the culture plate in a 37 ℃, 5% CO_2_ incubator for 24 h of cultivation. After methanol fixation and crystal violet (C0121, Beyotime, China) staining, cell counting was performed under an optical microscope (CX43, Olympus, Japan).

### Colorimetric assay

The levels of Fe²⁺, GSH-Px and GSH in the cells were detected using commercial colorimetric assay kits. The Mirco Cell Ferrous Ion Content Assay Kit (KTB1116) and the Micro GSH-Px Activity Assay Kit (KTB1640) were purchased from Abbkine (Wuhan, China), while the Reduced GSH Colorimetric Assay Kit (E-BC-K030-M) was obtained from Elabscience (Wuhan, China). Cells were collected and gently washed twice with cold PBS. Then, the cells were resuspended in the specific lysis buffer, centrifuged at 3000 rpm for 15 min in a 4 ℃, and the supernatant was collected. All procedures were strictly performed following the manufacturer’s instructions.

### Dual luciferase reporter assay

The wild-type (WT) sequence of the 3’UTR of the TAOK1 gene, which contains the miR-557 binding site, was cloned into the pmirGLO reporter plasmid (E1330, Promega, USA), and a mutant (MUT) plasmid with the binding site mutation was constructed. The aforementioned reporter plasmid was co-transfected with a luciferase control plasmid from the sea kidney into the experimental cells, and co-transfections were also performed with miR-557 mimics, inhibitors, or their negative controls respectively. After transfection for 48 h, the cells were lysed using the dual luciferase detection system (E1910, Promega, USA), and the activities of luciferase from firefly and sea kidney were measured successively.

### Statistical analysis

Data were analyzed using SPSS 26.0 and GraphPad Prism 9.0 software. The Chi-square test was used for the comparison of categorical variables, while Student’s t-test or one-way ANOVA was used for the comparison of continuous variables between groups. Survival analysis was performed using the Kaplan-Meier method to generate survival curves, and the Cox proportional hazards regression model was used to evaluate prognostic factors. Pearson correlation analysis was used to assess the correlation between miR-557 and TAOK1 expression levels. *P* < 0.05 was considered statistically significant.

## Results

### Expression of miR-557 in CC tissues and its clinical significance

**Fig. 1:**
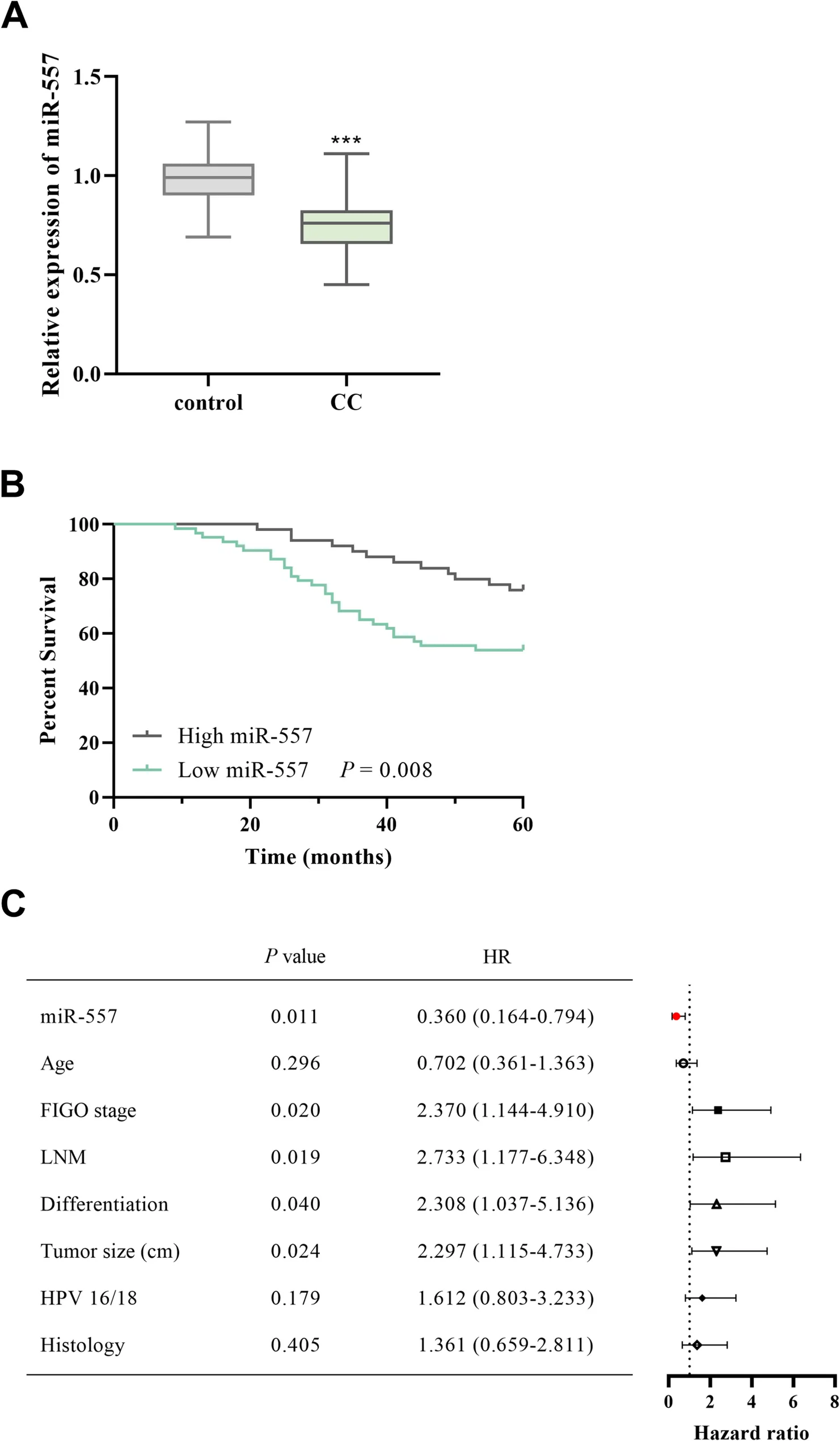
The expression and clinical significance of miR-557. **A** The expression of miR-557 in CC tissues. **B** Kaplan-Meier curves compare the survival differences among patients with CC in different groups. **C** Cox regression analysis evaluate the prognostic risk factors for CC. ***P < 0.001 vs. control group

The results of qRT-PCR revealed that miR-557 expression in 113 CC tissues was significantly lower compared to adjacent normal tissues (*P* < 0.001, Fig. 1A).

Patients were divided into high- and low-miR-557 expression groups based on the median level in tumor tissues, and clinical pathological characteristics were compared between the two groups. The data indicated that miR-557 expression was markedly correlated with FIGO stage (*P* = 0.006), LNM (*P* = 0.014), degree of differentiation (*P* = 0.034), and tumor size (*P* = 0.034, Table 1).

**Table 1:** Associations between miR-557 expression and clinical pathological characteristics

Characteristic	Case	miR-557 expression			*P* value
	(*n* = 113)	Low (*n* = 63)	High (*n* = 50)		
Age (years)					0.139
< 45	50 (44.25%)	24 (38.10%)		26 (52.00%)	
≥ 45	63 (55.75%)	39 (61.90%)		24 (48.00%)	
FIGO stage					0.006
I-II	70 (61.95%)	32 (50.79%)		38 (76.00%)	
III	43 (38.05%)	31 (49.21)		12 (24.00%)	
LNM					0.014
Negative	67 (59.29%)	31 (49.21%)		36 (72.00%)	
Positive	46 (40.71%)	32 (50.79%)		14 (28.00%)	
Differentiation					0.034
Poor	44 (38.94%)	30 (47.62%)		14 (28.00%)	
Moderate-high	69 (61.06%)	33 (52.38%)		36 (72.00%)	
Tumor size (cm)					0.034
< 2	62 (54.87%)	29 (46.03%)		33 (66.00%)	
≥ 2	51 (45.13%)	34 (53.97%)		17 (34.00%)	
HPV 16/18					0.091
Negative	60 (53.10%)	29 (46.03%)		31 (62.00%)	
Positive	53 (46.90%)	34 (53.97%)		19 (38.00%)	
Histology					0.466
Squamous cell carcinoma	82 (72.57%)	44 (69.84%)		38 (76.00%)	
Adenocarcinoma and others	31 (27.43%)	19 (30.16%)		12 (24.00%)	

*FIGO* International Federation of Gynecology and Obstetrics, *LNM* Lymph Node Metastasis, *HPV* Human Papillomavirus. *P* < 0.05 means a significant difference

K-M curve analysis demonstrated that the overall survival rate of patients with low miR-557 expression was markedly lower than that in the high-expression group (*P* = 0.008, Fig. 1B). Further Cox regression analysis revealed that in CC, FIGO stage (HR = 2.370, 95% CI: 1.144–4.910, *P* = 0.020), lymph node metastasis (HR = 2.733, 95% CI: 1.177–6.348, *P* = 0.019), tumor differentiation (HR = 2.308, 95% CI: 1.037–5.136, *P* = 0.040), and tumor size (HR = 2.297, 95% CI: 1.115–4.733, *P* = 0.024) were all independent prognostic risk factors, whereas miR-557 (HR = 0.360, 95% CI: 0.164–0.794, *P* = 0.011) acted as an independent protective factor (Fig. 1C). Moreover, miR-557 exhibited the strongest association with the prognosis of CC patients among all evaluated factors.

### The regulatory effect of miR-557 on CC cells

**Fig. 2:**
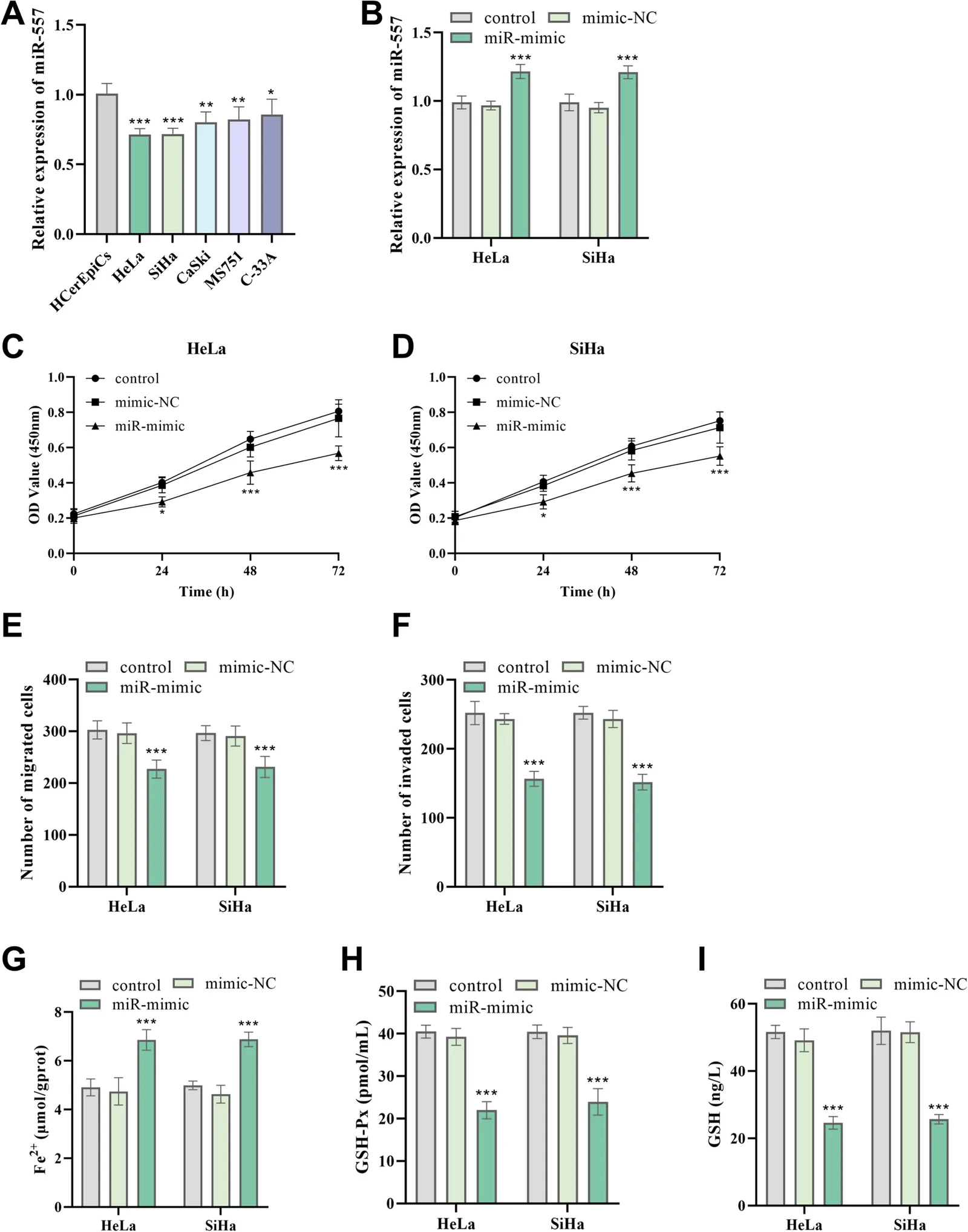
The regulatory effect of miR-557 on CC cells. **A** The expression of miR-557 in different CC cell lines. **B** The effect of miR-557 mimic on the expression levels of miR-557. **C-D**: The effect of miR-557 mimic on the proliferation activity of HeLa (**C**) and SiHa (**D**) cells. **E-F**: The effect of miR-557 mimic on the migration (**E**) and invasion(**F**) of CC cells. **G-I**: The effects of miR-557 mimic on the Fe^2+^ concentration (**G**), GSH-Px activity (**H**) and GSH level (**I**) in CC cells. *P < 0.05, **P < 0.01, ***P < 0.001 vs. HCerEpiCs (in A). *P < 0.05, **P < 0.01, ***P < 0.001 vs. mimic-NC (in B-I)

qRT-PCR results indicated that compared to the HCerEpiCs, miR-557 expression in five CC cell lines were all significantly downregulated: HeLa (*P* < 0.001), SiHa (*P* < 0.001), CaSki (*P* = 0.002), MS751 (*P* = 0.004), and C-33 A (*P* = 0.021, Fig. 2A). Based on this, the study selected the two most significantly downregulated cell lines, HeLa and SiHa, for the subsequent transfection experiments.

Following transfection with miR-557 mimic, miR-557 expression in HeLa and SiHa cells was substantially increased (*P* < 0.001, Fig. 2B), indicating that successful transfection was achieved for subsequent functional assays.

CCK-8 assay results confirmed that the proliferation of HeLa and SiHa cells was significantly reduced after transfection with miR-557 mimic (*P* < 0.001, Fig. 2C-D). Transwell assay results further indicated that overexpression of miR-557 markedly suppressed the migration and invasion abilities of HeLa and SiHa cells (*P* < 0.001, Fig. 2E-F).

Compared with the negative control group, transfection with miR-557 mimic significantly increased Fe²⁺ concentrations in HeLa and SiHa cells (*P* < 0.001, Fig. 2G). Additionally, GSH-Px and GSH levels in cells were notably decreased following transfection with the miR-557 mimic (*P* < 0.001, Fig. 2H-I).

### Expression of TAOK1 in CC tissues and cells

**Fig. 3:**
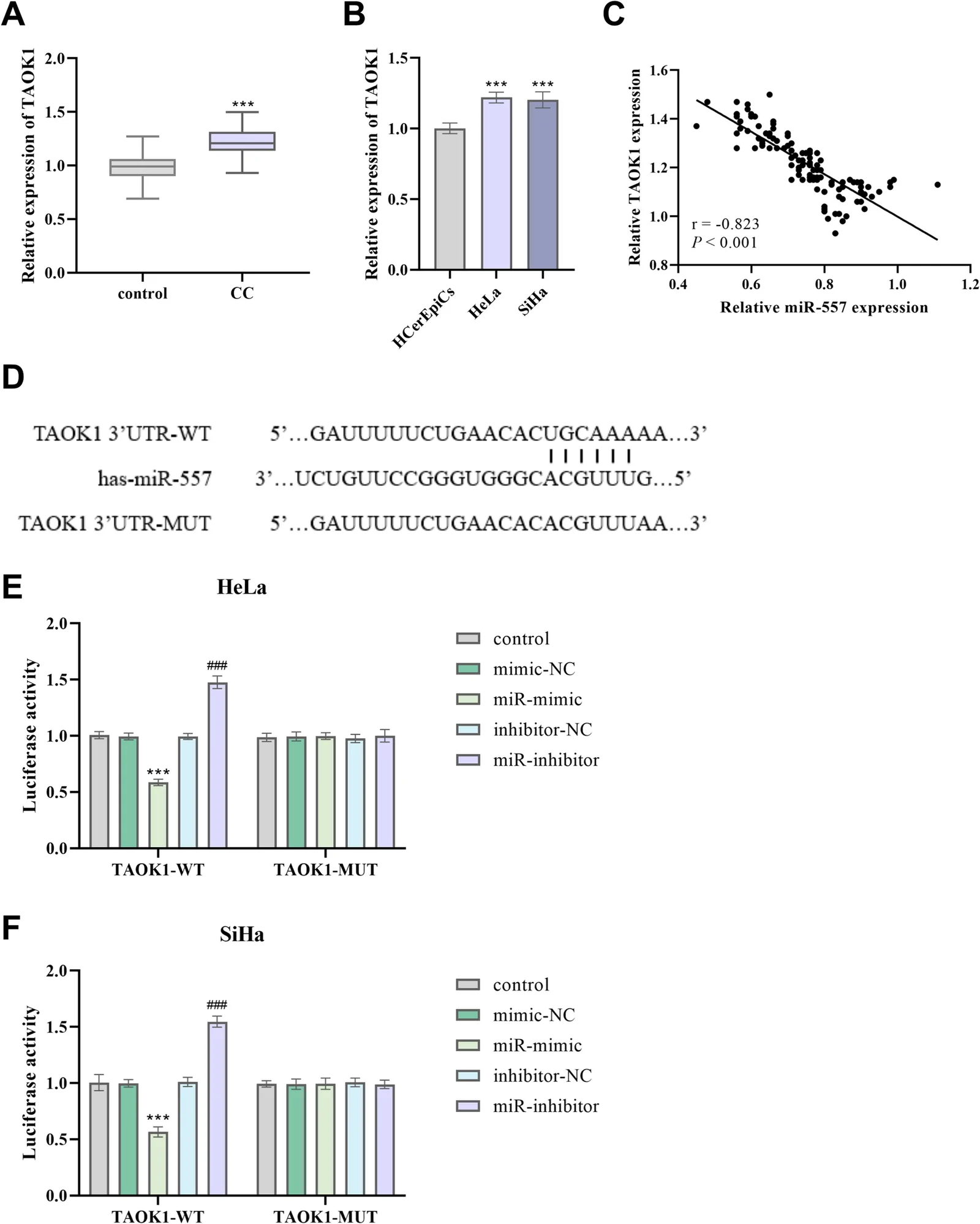
Expression of TAOK1 and verification of targeted binding. **A** The expression of TAOK1 in the CC tissue. **B** The expression of TAOK1 in CC cells. **C** The correlation between TAOK1 and the expression level of miR-557. **(D)** The target binding sites of TAOK1 and miR-557. **E-F**: Dual-luciferase reporter assay validated the targeting interaction between miR-557 and TAOK1 in HeLa (**E**) and SiHa (**F**) cells. ***P < 0.001 vs. control group (in A, B). ***P < 0.001 vs. mimic-NC; ^###^*P* < 0.001 vs. inhibitor-NC (in E, F)

Compared with adjacent normal tissue, TAOK1 expression was considerably elevated in CC tissue (*P* < 0.001, Fig. 3A). Furthermore, TAOK1 also exhibited significantly higher expression in the CC cell lines HeLa and SiHa (*P* < 0.001, Fig. 3B). Pearson correlation analysis was performed on the expression levels of miR-557 and TAOK1. The results revealed a significant negative correlation between the two (*r* = -0.823, Fig. 3C).

### Verification of targeted binding between miR-557 and TAOK1

Using the online database TargetScan, we predicted potential binding sites between miR-557 and TAOK1 (Fig. 3D). To validate the direct target relationship between miR-557 and TAOK1, a dual luciferase reporter assay was performed. The results confirmed that, in both HeLa and SiHa cells, the miR-557 mimic significantly inhibited the luciferase activity of the TAOK1-WT reporter gene (*P* < 0.001). Conversely, its inhibitor significantly increased this activity (*P* < 0.001). However, neither had a significant effect on the activity of the TAOK1-MUT reporter gene (*P* > 0.05, Fig. 3E-F).

### Mechanism of the miR-557/TAOK1 axis in CC progression

**Fig. 4:**
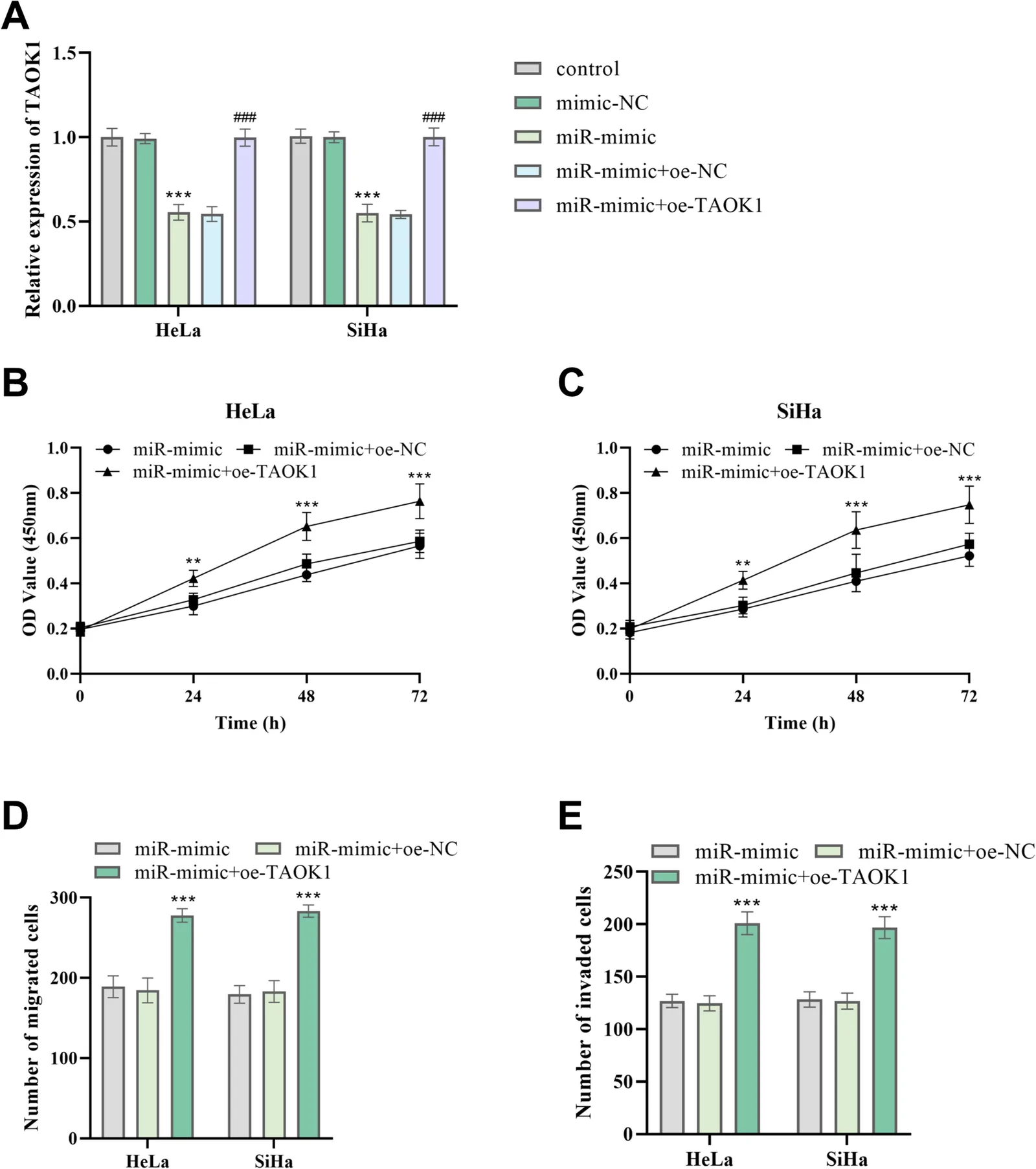
miR-557 regulates CC progression by targeting TAOK1. **A.** The regulatory effect of miR-557 on TAOK1 expression. **B-C**: The effect of miR-557 targeting TAOK1 on the proliferation activity of HeLa (**B**) and SiHa (**C**) cells. **D-E**: The effect of miR-557 targeting TAOK1 on the migration (**D**) and invasion (**E**) of CC cells. ***P < 0.001 vs. mimic-NC; ^###^*P* < 0.001 vs. miR-mimic + oe-NC (in A). **P < 0.01, ***P < 0.001 vs. miR-mimic + oe-NC (in B-E)

In HeLa and SiHa cells, the miR-557 mimic significantly suppressed TAOK1 expression (*P* < 0.001), whereas after transfection with the oe-TAOK1 vector significantly increased TAOK1 expression (*P* < 0.001, Fig. 4A).

Rescue assay results demonstrated that reintroduction of TAOK1 in CC cells overexpressing miR-557 significantly reversed the inhibitory effect of miR-557 on malignant phenotypes. Specifically, TAOK1 overexpression not only significantly enhanced the proliferative activity of CC cells (*P* < 0.001, Fig. 4C-D), but also markedly increased their migration and invasive capacities (*P* < 0.001, Fig. 4E-F).

**Fig. 5:**
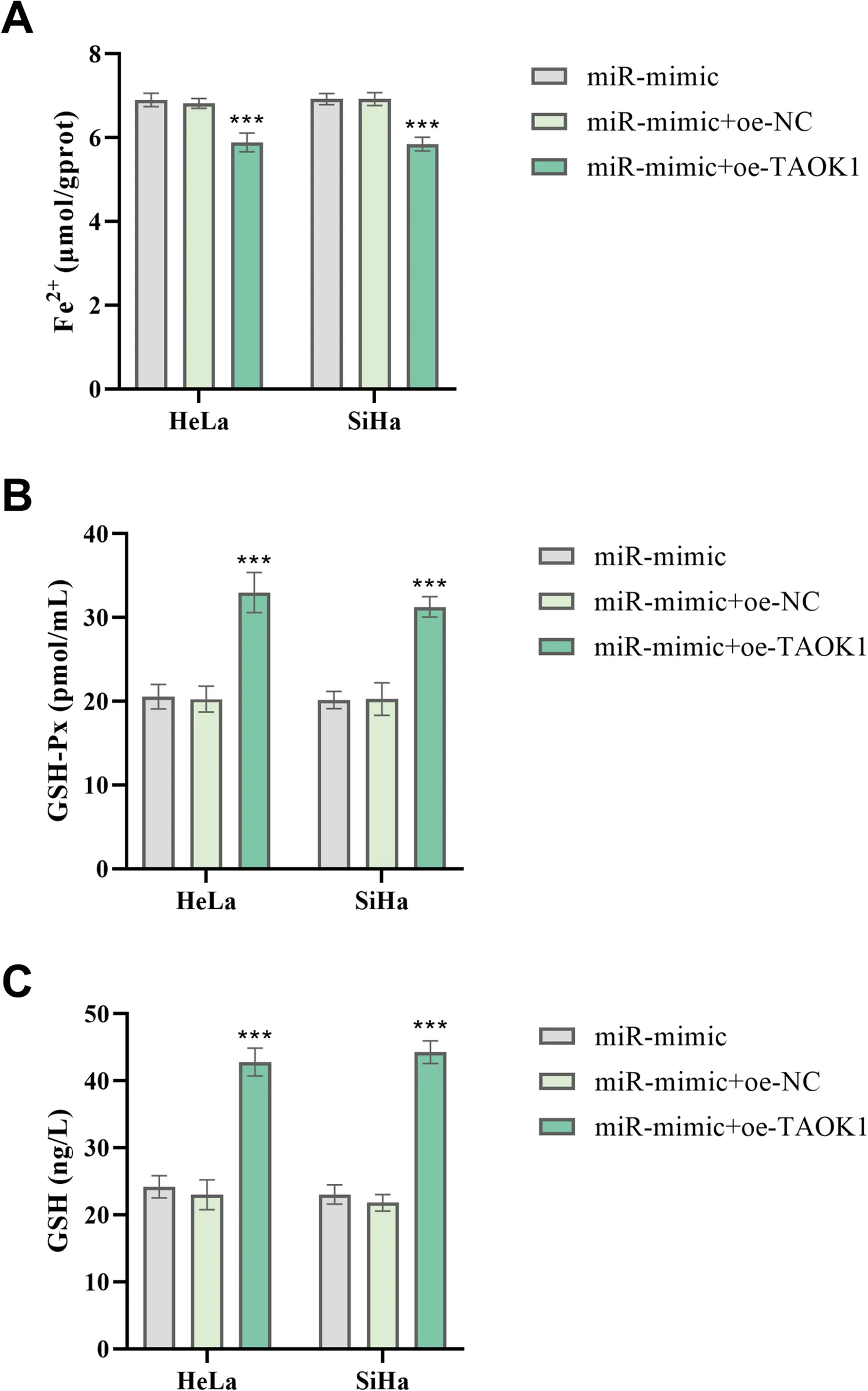
miR-557 targeting of TAOK1 modulates intracellular redox homeostasis in CC cells. **A-C**: The effect of miR-557 targeting TAOK1 on iron ions concentration (**A**), GSH-Px activity (**B**) and GSH levels (**C**) in CC cells. ***P < 0.001 vs. miR-mimic + oe-NC

Furthermore, co-overexpression of TAOK1 rescued the abnormalities induced by the miR-557 mimic in CC cells. TAOK1 overexpression not only significantly attenuated the miR-557-induced elevation of Fe²⁺ (*P* < 0.001, Fig. 5A) but also restored the suppressed GSH-Px activity and GSH levels (*P* < 0.001, Fig. 5B-C).

## Discussion

The harm of CC lies not only in the impact of the disease itself on patients but also in its high risk of recurrence and poor prognosis [13]. Patients with advanced and recurrent disease often face significant challenges, including limited treatment options and a markedly reduced five-year survival rates [14]. Therefore, identifying novel biomarkers is currently key to improving the prognosis of CC. It has been reported that many miRNAs are dysregulated in CC and are related to the development of the disease [15, 16]. Consistent with these reports, miR-557 shows a significant downward trend in CC [5], which strongly supports the findings of this study and suggests that miR-557 may be involved in the progression of CC.

In the clinical diagnosis, treatment, and prognosis assessment of CC, the FIGO stage, LNM, tumor differentiation grade, and tumor size are four critical influencing factors. The FIGO stage serves as the core system for assessing disease extent, directly guiding treatment strategy formulation. Later stages typically indicate more complex diseases and are associated with a poorer prognosis [17]. LNM, as a key indicator of tumor spread, significantly influences treatment decisions and patient survival outcomes [18]. Tumor differentiation and size, as core pathological features, jointly determine tumor biological behavior and invasive potential [19, 20]. The present study found that miR-557 expression levels were significantly correlated with the aforementioned clinicopathological parameters, suggesting a strong link between miR-557 and CC progression. Importantly, this correlation with deteriorating clinical parameters was further reflected in patient prognosis. Cox regression analysis identified miR-557 as an independent protective factor for overall survival in patients with CC. This finding implies that low miR-557 expression is associated with a higher risk of poor prognosis, further reinforcing its potential role as a clinically meaningful prognostic biomarker in CC progression.

Based on this, we conducted cellular experiments to further investigate the regulatory mechanism of miR-557. The proliferation, migration, and invasion of cancer cells represent core biological behaviors in malignant tumor progression. Collectively, these processes drive tumorigenesis and metastasis, directly impacting patient prognosis [21, 22]. Our results demonstrated that upregulating miR-557 significantly suppressed these malignant phenotypes in CC cells. This observation aligns with the tumor-suppressive role of miR-557 reported in other cancer types [6], but more importantly, it provides the functional evidence that miR-557 directly restricts the aggressive behavior of CC cells. These findings support the notion that miR-557 may function as a potential molecular target for therapeutic intervention in CC.

Ferroptosis, as a novel type of iron-dependent programmed cell death. It has become a leading research hotspot in the field of cancer studies [23, 24]. Recent studies have revealed that multiple miRNAs can precisely regulate the core pathways of ferroptosis. Through this regulation, they modulate the oncogenic phenotypes of cancer cells and emerge as critical molecules involved in cancer progression [25]. The intracellular concentration of ferrous ions, the activity of GSH-Px, and the level of GSH are all important components of the key regulatory links in ferroptosis [26]. In this study, we found that upregulation of miR-557 markedly elevated the concentration of ferrous ions in CC cells. At the same time, it reduced GSH-Px activity and GSH levels. These findings suggest that overexpression of miR-557 may disrupt iron metabolism and elevate oxidative stress levels in CC cells. Consequently, the ability of cancer cells to maintain their normal active state is impaired, leading to inhibition of their growth and proliferation.

Numerous studies have indicated that TAOK1 promotes tumor development in multiple cancers, and its expression levels are significantly correlated with patient prognosis [12]. For example, in non-small cell lung cancer, TAOK1 promotes tumor cell proliferation and metastasis [10]. In the present study, we confirmed through dual-luciferase reporter and rescue experiments that TAOK1 is a direct target of miR-557 in CC cells, and that TAOK1 overexpression markedly attenuated the inhibitory effects of miR-557 on malignant phenotypes, suggesting that miR-557 regulates CC progression by modulating TAOK1 expression. Mechanistically, TAOK1 belongs to the MAP3K family and serves as an upstream activator of the p38 MAPK and JNK signaling pathways [27]. As members of the MAPK cascade, TAOKs phosphorylate and activate MKK3/6, which in turn activate p38 MAPK; TAOK1 and TAOK2 also activate the JNK cascade through MKK4/7 [28]. Both the p38 and JNK pathways are well-established regulators of oxidative stress and cell death [29]. Activation of p38 MAPK can modulate intracellular reactive oxygen species (ROS) levels and glutathione metabolism, while JNK signaling has been shown to promote apoptosis and ferroptosis under certain conditions [30]. Given the established role of TAOK1 as an upstream activator of these pathways, we speculate that the miR-557/TAOK1 axis may exert its pro-ferroptosis effects by modulating p38/JNK-mediated oxidative stress responses in CC cells. Our findings demonstrate that miR-557 suppresses malignant progression in CC cells by targeting TAOK1, leading to ferrous ion accumulation and elevated oxidative stress levels, which together suggest the induction of ferropototic stress. Future studies are warranted to directly investigate whether p38/JNK signaling mediates the pro-ferroptosis effects of the miR-557/TAOK1 axis in CC.

However, this study still has certain limitations. The study population samples were all derived from a single medical center with a limited sample size; thus, future validation should be conducted using multi-center, large-scale prospective cohort studies. Additionally, this study did not employ rescue experiments with ferroptosis-specific inhibitors to definitively confirm the mode of cell death, nor did we directly investigate the downstream signaling pathways (e.g., p38 MAPK or JNK) through which the miR-557/TAOK1 axis may regulate ferroptosis. Therefore, whether the miR-557/TAOK1 axis exerts its tumor-suppressive function by directly inducing ferroptosis, and whether this effect is mediated via p38 or JNK pathways, remain to be further validated in subsequent experiments.

## Conclusion

In summary, miR-557 is significantly downregulated in CC and exerts a tumor-suppressive effect. Low expression of miR-557 is associated with a poor prognosis in CC patients and may serve as a potential biomarker for CC. Mechanistically, miR-557 inhibits the malignant phenotype of CC cells by suppressing TAOK1 expression, thereby disrupting intracellular homeostasis and ultimately suppressing the development of CC.

## Data Availability

The datasets used and/or analysed during the current study are available from the corresponding author on reasonable request.
